# Children’s propensity to consume sugar and fat predicts regular alcohol consumption in adolescence

**DOI:** 10.1017/S1368980018001829

**Published:** 2018-08-24

**Authors:** Kirsten Mehlig, Leonie H Bogl, Monica Hunsberger, Wolfgang Ahrens, Stefaan De Henauw, Isabel Iguacel, Hannah Jilani, Dénes Molnár, Valeria Pala, Paola Russo, Michael Tornaritis, Toomas Veidebaum, Jaakko Kaprio, Lauren Lissner

**Affiliations:** 1 Section for Epidemiology and Social Medicine, Institute of Medicine, Sahlgrenska Academy, University of Gothenburg, Box 453, SE-405 30, Gothenburg, Sweden; 2 Department of Public Health, University of Helsinki, Helsinki, Finland; 3 Leibniz Institute for Prevention Research and Epidemiology – BIPS, Bremen, Germany; 4 Institute of Statistics, University of Bremen, Bremen, Germany; 5 Department of Public Health, Ghent University, Ghent, Belgium; 6 Growth, Exercise, Nutrition and Development (GENUD) Research Group, University of Zaragoza, Zaragoza, Spain; 7 Department of Pediatrics, Medical School, University of Pécs, Pécs, Hungary; 8 Epidemiology and Prevention Unit, Fondazione IRCCS Istituto Nazionale dei Tumori, Milan, Italy; 9 Institute of Food Sciences, CNR, Avellino, Italy; 10 Research and Education Institute of Child Health, Strovolos, Cyprus; 11 National Institute for Health Development, Tallinn, Estonia; 12 Institute for Molecular Medicine FIMM, University of Helsinki, Helsinki, Finland

**Keywords:** Alcohol consumption in adolescence, Sugar and fat intake in childhood, Childhood risk factors, Cohort study

## Abstract

**Objective:**

The present study investigated the association between sugar and fat intake in childhood in relation to alcohol use in adolescence. We hypothesized that early exposure to diets high in fat and sugar may affect ingestive behaviours later in life, including alcohol use.

**Design/Setting/Subjects:**

Children from the European IDEFICS/I.Family cohort study were examined at ages 5–9 years and followed up at ages 11–16 years. FFQ were completed by parents on behalf of children, and later by adolescents themselves. Complete data were available in 2263 participants. Children’s propensities to consume foods high in fat and sugar were calculated and dichotomized at median values. Adolescents’ use of alcohol was classified as at least weekly *v*. less frequent use. Log-binomial regression linked sugar and fat consumption in childhood to risk of alcohol use in adolescence, adjusted for relevant covariates.

**Results:**

Five per cent of adolescents reported weekly alcohol consumption. Children with high propensity to consume sugar and fat were at greater risk of later alcohol use, compared with children with low fat and low sugar propensity (relative risk=2·46; 95 % CI 1·47, 4·12), independent of age, sex and survey country. The association was not explained by parental income and education, strict parenting style or child's health-related quality of life and was only partly mediated by sustained consumption of sugar and fat into adolescence.

**Conclusions:**

Frequent consumption of foods high in fat and sugar in childhood predicted regular use of alcohol in adolescence.

Early initiation and habituation to alcohol can change the developing brain and potentially increase the likelihood of addiction to alcohol and other substances^(^ ^1^ ^–^ ^3^ ^)^. Although alcohol use and alcoholism have strong hereditary origins, environmental factors may independently affect risk or modify genetic and other familial influences^(^ ^4^ ^–^ ^7^ ^)^. In this context, diet is a potentially modifiable early-life factor that might have an influence on later alcohol use. Animal experiments suggest that excessive sugar consumption can lead to addictive behaviour like the consumption of alcohol or drugs^(^ ^6^ ^–^ ^8^ ^)^, and it has been proposed that sugar addiction is also observed in man^(^ ^9^ ^–^ ^12^ ^)^. This claim was criticized because sugar consumption is associated with reward and craving, but other symptoms of addiction such as tolerance or withdrawal are less clear^(^ ^13^ ^)^. It has also been argued that the compulsive behaviour observed in rodents was not triggered by sucrose itself but by the palatability of it, particularly in combination with fat^(^ ^13^ ^,^ ^14^ ^)^. Although there is evidence for associations between sweet preference and alcohol dependence in human subjects^(^ ^9^ ^,^ ^15^ ^)^, and between fat and ethanol intake in rodents^(^ ^16^ ^)^, addictive-like behaviour may manifest itself in overconsumption of preferred foods rather than of a single nutrient^(^ ^14^ ^)^. These overconsumed foods are typically high in sugar, fat and salt, and this pattern of consumption has been summarized as ‘food addiction’^(^ ^17^ ^,^ ^18^ ^)^. Hebebrand *et al*. highlight the behavioural aspects of overeating and provide evidence for ‘eating addiction’ associated with palatable foods high in fat and sugar in contrast to purely substance-related addictive-like behaviour^(^ ^19^ ^)^. Thus, extreme eating behaviours may be indicative of loss of control and craving, which are central features of addiction, and excessive consumption of palatable foods that are high in fat and sugar may serve as one such signal.

Identification of modifiable environmental factors early in life is central to prevention of later alcohol use and abuse. The European IDEFICS/I.Family cohort study was designed to observe developments and changes in health-related behaviours between childhood and adolescence^(^ ^20^ ^)^. The present study covers a variety of geographical areas with distinctly different alcohol and food cultures, and has collected information on critical covarying factors such as parenting style, parental alcohol use and socio-economic conditions. Our aim is to explore the relationship between sugar and fat consumption in childhood and subsequent alcohol intake in adolescence, with emphasis on measured confounding and mediating factors.

## Participants and methods

The IDEFICS (Identification and prevention of Dietary- and lifestyle-induced health EFfects In Children and infantS) study was initiated in 2006 with survey centres in eight European countries (Belgium, Cyprus, Estonia, Germany, Hungary, Italy, Spain and Sweden). A total of 16 224 children were examined at baseline (2007–08; ages 2–9 years), providing data on diet, lifestyle and anthropometry^(^ ^21^ ^)^. The subsequent I.Family study included a longitudinal follow-up of the baseline cohort (2013–14; ages 8–16 years) as well as information on family members^(^ ^20^ ^)^. At the time of the first follow-up (2009–10), the participation rate was 68 %, and attrition was associated with overweight, lower well-being scores and lower parental education and income^(^ ^22^ ^)^. The final follow-up had a participation rate of 55 %, indicating further attrition^(^ ^20^ ^)^.

### Assessment of exposure and outcome

Children's dietary exposure to sugar and fat at baseline was based on answers to a forty-three-item FFQ that was filled in by 15 168 parents^(^ ^21^ ^,^ ^23^ ^–^ ^26^ ^)^. The consumption frequency of foods high in sugar was calculated in times per week, including fruit juice, jam, honey, chocolate or nut-based spread, and sweet snacks, as well as the following items if they contained added sugar: fresh fruit, drinks, milk, yoghurt and breakfast cereals. A propensity to consume foods rich in sugar was defined as the weekly frequency of foods high in sugar divided by the total frequency of all foods assessed in this FFQ. For instance, a value of 25 % for sugar propensity means that a quarter of all food frequencies reported were from sugar-rich foods. A similarly defined fat propensity was based on intake of fried potatoes, high-fat milk and yoghurt, fried fish, meat products, fried eggs, mayonnaise, cheese, chocolate or nut-based spread, butter and margarine, nuts, savoury snacks, chocolate, cake and biscuits, and ice cream. These food groups as well as the propensities have shown relative validity in relation to repeated 24 h recalls^(^ ^26^ ^)^. In spite of some common food items, the mutual correlation between sugar and fat propensities was small in magnitude (*r*=0·12, *P*<0·001). In addition to continuous variables we examined categories of high sugar or high fat propensity by dichotomizing the respective propensities at the median percentage. This was done to facilitate interaction and mediation analyses. Finally, a composite indicator was defined comparing the category of both high sugar and high fat propensity with the combination of the three remaining categories: high sugar and low fat, low sugar and high fat, and low sugar and low fat. Sensitivity analyses based on tertiles were performed to demonstrate independence of results on specific cut-off points. Six years after the baseline examination, the adolescents (now aged 11–16 years) were asked to complete an FFQ with questions almost identical to the baseline FFQ, and propensities were calculated that are comparable to those at baseline. The FFQ for adolescents also included one item for alcoholic beverages^(^ ^27^ ^)^. Use of alcohol was defined as at least weekly consumption *v.* less than weekly use, including no use. Dietary information in both childhood and adolescence was complete in 2263 individuals.

### Assessment of confounding variables

To adjust for weight status, we included age- and sex-standardized *Z*-scores of BMI measured at baseline and at follow-up, and overweight at baseline was defined using International Obesity Task Force cut-off points^(^ ^28^ ^)^. Parental education was classified based on the International Standard Classification of Education^(^ ^29^ ^)^ and dichotomized at medium level (post-secondary *v.* less education) reflecting the maximum educational level of both parents. Family income was dichotomized into high *v.* medium or low income based on standardized country-specific income categories^(^ ^30^ ^)^. Also at baseline, parents were asked to what extent they agreed with the statement ‘At our home it is laid down quite clearly what is allowed and what is not’ and parenting style was categorized into ‘strict’, somewhat strict’ and ‘permissive or rather permissive’^(^ ^31^ ^)^. A psychological quality-of-life score (HRQoL) was defined based on four dimensions of the parent- and self-report versions of the health-related quality-of-life KINDL-R questionnaire^(^ ^32^ ^)^ (emotional well-being, self-esteem, relations with family, relations with peers) for children and adolescents, respectively, with a range of 12–48, and higher values indicating better HRQoL. At the time of the follow-up, parents were asked to complete the same FFQ as the adolescents and the number of drinks per week was recorded for the parent who completed the FFQ, or the maximum number if both parents answered. In addition to the HRQoL, teens answered questions on their tendency to rash action that were combined into a score (range 12–48) with higher values indicating higher impulsivity^(^ ^33^ ^)^. Both HRQoL and impulsivity scores were included as potential correlates of exposure and outcome variables.

### Statistical methods

Descriptive statistics are presented as mean and standard deviation for continuous variables and as frequency and percentage for binary variables. Alcohol consumption at follow-up in relation to baseline predictors was assessed using log-binomial regression^(^ ^34^ ^)^; the result is given in terms of the relative risk (RR) for alcohol consumption together with the 95 % confidence interval. Exposure variables, namely sugar and fat propensities at baseline, were analysed as (i) continuous variables and categorical variables at (ii) the median or (iii) tertile values. The basic model for alcohol consumption as a function of sugar and fat propensities included age and sex as fixed effects as well as country as a random effect, to account for the low prevalence of ≥weekly alcohol consumption in this age group. Additional models tested the impact of potential confounders, as well as the interaction between high sugar and high fat propensity. To test for country-specific associations between alcohol consumption and earlier dietary exposure we added an interaction term between exposure and country as a random effect. Potential collinearity between predictors was assessed by calculating the variance inflation factor using a linear model for the prevalence of alcohol consumption. All regression models presented here showed variance inflation factor values less than 2. The log-binomial regression models included a random effect to allow for overdispersion and goodness-of-fit was assessed in terms of the scaled Pearson statistics, with values close to 1 indicating a good fit. Complete case analysis was applied for covariates with missing values (<10 % of total sample size, except for parental alcohol use which was available for 1627 adolescents only). Mediation analysis was performed to examine to what extent the prospective association between a preference for foods high in fat and sugar in childhood and alcohol consumption in adolescence was due to sustained consumption of these foods in adolescence. The mediation analysis was conducted using a SAS macro published by Valeri and VanderWeele^(^ ^35^ ^)^ consisting of two regression steps assessing: (i) the association of alcohol use at follow-up with diet at baseline and at follow-up; and (ii) the association between diet at follow-up and diet at baseline. Statistical analyses were performed using the statistical software package SAS version 9.4. Statistical significance was set at *P*<0·05 (two-sided tests).

## Results

### Descriptive characteristics of the sample at baseline and at follow-up

At baseline, the children were 5–9 years old and 51 % of them were girls (*n* 1155). Weight status in terms of age- and sex-specific BMI *Z*-score did not differ between time points. Half of the families reported a high level of parental education and 37 % reported high income. Compared with childhood, adolescents reported higher fat and lower sugar propensities, and 5 % reported weekly alcohol consumption or more. Additional characteristics for potential confounders or mediators of the association between diet and alcohol consumption are given in Table 1.

**Table 1:** Participant characteristics at baseline and follow-up among children from eight European countries examined at ages 5–9 years and followed up at ages 11–16 years (*n* 2263 if not indicated otherwise), IDEFICS/I.Family cohort study

	Baseline		6-year follow-up	
	Mean or *n*	sd or %	Mean or *n*	sd or %
Children/teens				
Age (years)	7·51	0·77	13·37	0·74
BMI *Z*-score	0·52	1·18	0·53	1·25
Sugar propensity (%)	25·0	11·4	22·6	10·0
Fat propensity (%)	25·3	9·5	27·7	10·1
HRQoL†	39·5	4·7	38·4	5·2
Impulsivity score†	–	–	24·9	7·5
Weekly use of alcohol, *n* and %	–	–	107	5
Current smoking†, *n* and %	–	–	45	2
Parents				
High education†, *n* and %	1106	49	–	–
High income†, *n* and %	762	37	–	–
Parenting style†, *n* and %				
Permissive	277	13	–	–
Somewhat strict	1118	52	–	–
Strict	745	35	–	–
No. of alcoholic drinks per week†	–	–	1·6	3·5

IDEFICS, Identification and prevention of Dietary- and lifestyle-induced health EFfects In Children and infantS; HRQoL, health-related quality of life. Data presented are mean and sd unless indicated otherwise.

† *n*<2263 due to missing values (cf. online supplementary material, Tables S1 and S2).

### Cross-sectional characteristics associated with sugar and fat propensities, and with alcohol consumption

Sugar and fat propensities were dichotomized at their median values of 24·0% and 24·6%, respectively, to examine characteristics of high sugar or high fat propensity (see online supplementary material, Table S1). There were no differences by age or sex between categories of sugar or fat propensity in childhood. Children with high sugar or high fat propensity had lower BMI *Z*-score compared with reference. High sugar propensity was associated with lower parental education and a less strict parenting style, while high fat propensity was associated with lower HRQoL. Compared with overall averages, children in Italy, Belgium and Germany were most likely to consume foods high in sugar or fat, while children from the Swedish survey centre were least likely to do so (data not shown).

Follow-up characteristics are presented by alcohol consumption status (see online supplementary material, Table S2). Participants were between 11·0 and 16·2 years old, with similar age distribution in both alcohol categories. Boys were more likely to report weekly alcohol intake than girls. Alcohol consumption was associated with higher values of concurrent sugar and fat propensities, but not with weight status. Adolescents consuming alcohol showed lower HRQoL, higher impulsivity and were more likely to report smoking than their non-drinking peers. Parents of alcohol-consuming adolescents reported higher alcohol consumption compared with parents of adolescents who did not drink (point-biserial correlation coefficient=0·06, *P*=0·01). The country-specific prevalence of ≥weekly alcohol consumption was 7 % (Italy), 6 % (Cyprus), 5 % (Hungary), 4 % (Belgium and Estonia), 2 % (Germany and Spain) and 1 % (Sweden).

### Consumption of sugar and fat in childhood and alcohol consumption in adolescence

A higher value of sugar propensity in childhood predicted use of alcohol in adolescence, RR=1·20 (95 % CI 1·01, 1·43) for 1 SD, adjusted for age, sex and country. The corresponding association between fat propensity and alcohol use was somewhat stronger than that for sugar, RR=1·27 (95 % CI 1·09, 1·49) per 1 sd. In a mutually adjusted model, fat propensity was independently predictive of alcohol use, RR=1·25 (95 % CI 1·06, 1·47), but sugar propensity was not, RR=1·16 (95 % CI 0·98, 1·38). A prediction of alcohol use was also observed for the dichotomized sugar and fat propensities, RR=1·62 (95 % CI 1·11, 2·37) for high sugar and RR=1·78 (95 % CI 1·22, 2·59) for high fat propensity, and both associations were observed in the mutually adjusted model, RR=1·54 (95 % CI 1·06, 2·26) and RR=1·71 (95 % CI 1·17, 2·49), respectively.

Table 2 shows the risk of alcohol use as a function of dichotomized propensities including their interaction. The risk of alcohol consumption was more than doubled for the combination of high sugar and high fat propensity compared with the combination of low sugar and low fat propensity (Table 2A), but the interaction between high fat and high sugar consumption was not statistically significant (*P*=0·4). The association between categories of fat and sugar propensity and later alcohol use was independent of baseline measures of HRQoL, parental education, income and parenting style (Table 2B). Among the latter, only HRQoL was significantly associated with alcohol in the multivariable model, such that higher values were protective for later alcohol use, RR=0·81 (95 % CI 0·67, 0·98) per 1 sd. High parental income at baseline was associated with reduced risk of alcohol use in adolescence, RR=0·55 (95 % CI 0·34, 0·89), adjusted for diet, age, sex and country. Also, a stricter parenting style predicted less alcohol use: RR=0·69 (95 % CI 0·43, 1·13) and RR=0·56 (95 % CI 0·32, 0·97) for somewhat strict and strict *v*. permissive, respectively, adjusted for diet, age, sex and country.

**Table 2:** Associations between sugar and fat propensity at baseline and alcohol consumption in adolescence at follow-up among children from eight European countries examined at ages 5–9 years and followed up at ages 11–16 years (*n* 2263 if not indicated otherwise), IDEFICS/I.Family cohort study

	Categories of dichotomized sugar and fat propensities			
	Low sugar+ low fat	High sugar+ low fat	Low sugar+ high fat	High sugar+ high fat
Total *n*	619	511	514	619
No. of alcohol consumers	19	21	21	46
A. Adjusted for age, sex, country				
RR	1·00	1·27	1·40	2·46***
95 % CI	Ref.	0·70, 2·29	0·77, 2·54	1·47, 4·12
B. Further adjusted for HRQoL, parental income, education, parenting style†				
RR	1·00	1·25	1·36	1·79*
95 % CI	Ref.	0·68, 2·29	0·74, 2·49	1·03, 3·11

IDEFICS, Identification and prevention of Dietary- and lifestyle-induced health EFfects In Children and infantS; RR, relative risk; HRQoL, health-related quality of life; Ref., reference category. *P<0·05, ***P<0·001.

† *n* 1931 due to missing values for covariates.

### Sensitivity analyses

**Fig. 1:**
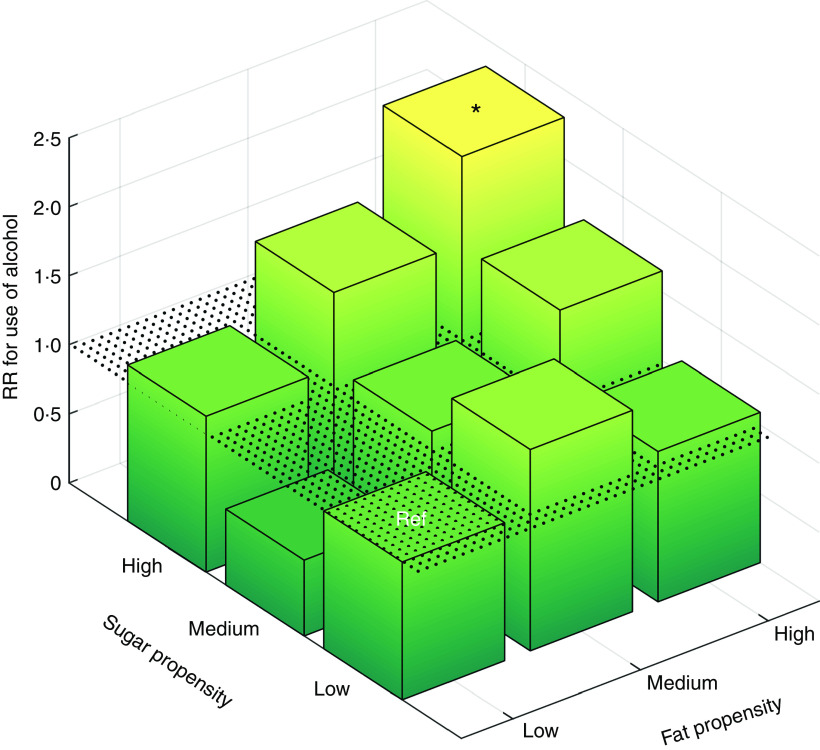
(colour online) Relative risk (RR) for use of alcohol in adolescence by sugar and fat propensity tertiles and their combinations at baseline (log-binomial regression adjusted for age, sex and country) among children from eight European countries examined at ages 5–9 years and followed up at ages 11–16 years (*n* 2263), IDEFICS/I.Family cohort study. *P<0·05 (Ref, reference category (lowest tertile in sugar propensity and in fat propensity); IDEFICS, Identification and prevention of Dietary- and lifestyle-induced health EFfects In Children and infantS)

Figure 1 illustrates the risk of alcohol use as a function of tertiles of sugar and fat propensities. Being in the combination of highest tertiles more than doubled the risk of alcohol use, RR=2·30 (95 % CI 1·16, 4·57) compared with reference. The associations of sugar and fat propensities with alcohol use did not differ by sex, country or other covariates listed in Table 1 (see online supplementary material, Tables S3 and S4). Baseline BMI *Z*-score did not predict later alcohol use, RR=0·98 (95 % CI 0·84, 1·15) adjusted for diet, age, sex and country, and the positive association between the combination of high sugar and high fat propensity with alcohol consumption was observed in both overweight and non-overweight children (Table S3). Finally, parental alcohol consumption was positively associated with adolescents’ alcohol consumption but not with the children’s diet at baseline or at follow-up. Consequently, adjusting for parental alcohol consumption did not change the associations between childhood diet and later alcohol consumption reported in Table 2.

### Is the prospective association between diet and alcohol mediated by diet at follow-up?

Table 2 showed that the combination of high sugar and high fat propensity was the strongest predictor of later alcohol consumption. For the purpose of mediation analysis, we defined a binary variable distinguishing the combination of high sugar and high fat propensity from all other categories combined, both for diets in childhood and in adolescence (Table 2). Both dietary indicators were associated with alcohol consumption. However, mediation analysis showed that the direct effect of diets high in fat and sugar in childhood on later alcohol use was stronger than the indirect effect through sustained preference for these foods in adolescence (Table 3).

**Table 3:** Mediation analysis: decomposition of the total effect of high sugar and high fat propensity at baseline on subsequent alcohol consumption into direct and indirect effects among children from eight European countries examined at ages 5–9 years and followed up at ages 11–16 years (*n* 2256), IDEFICS/I.Family cohort study

	RR	95 % CI
Direct effect of (a) diet at baseline on (b) alcohol at follow-up	1·78**	1·22, 2·60
Indirect effect via correlation of (c) diet at follow-up with (a) and (b)	1·12**	1·04, 1·21
Total effect via both pathways	1·99***	1·37, 2·90

IDEFICS, Identification and prevention of Dietary- and lifestyle-induced health EFfects In Children and infantS; RR, relative risk. **P<0·01, ***P<0·001 (adjusted for sex of the child and survey country).

## Discussion

In the European, multicentre, IDEFICS/I.Family cohort study we observed that children consuming sugar-rich or high-fat foods were at higher risk of alcohol consumption in adolescence. The associations were independent of age, sex and country, in spite of notable country differences in both exposure and outcome, and of male teenagers being more likely to report alcohol consumption. The highest risk for later alcohol consumption was observed for the combination of above-median sugar and above-median fat propensity, compared with all other combinations. An analysis based on tertiles of sugar and fat propensities confirmed the independence of the result on specific cut-off points. The associations between dietary preferences in childhood and later alcohol use were not explained by strict parenting style, which does not support the assumption that parents restricting their children’s sugar and fat intake would later limit their alcohol consumption. The results were not confounded by health-related quality of life, parental education and income, or parental alcohol use. Although the prevalence of high sugar and high fat consumption did not decline between childhood and adolescence, mediation analysis showed that the direct effect of sugar and fat consumption in childhood was larger than the indirect effect mediated through sustained consumption of sugar and fat into adolescence.

Adolescence represents a period of unique vulnerability for developing addiction^(^ ^4^ ^)^, with both parents and peers acting as role models and contributing to risk or resilience^(^ ^5^ ^,^ ^36^ ^,^ ^37^ ^)^. It is a challenge in observational epidemiology to distinguish the effect of an exposure such as dietary preference in childhood from covarying influences such as parenting style or socio-economic factors. On the family level, high parental income was associated with reduced risk of adolescent alcohol consumption, but the risk due to above-median values for sugar and fat propensity was not explained by parental income. Given the strong inter-country differences in both dietary preferences and alcohol consumption, it is remarkable that their mutual association did not differ by country. The survey centre in southern Italy showed the highest prevalence of alcohol consumption among adolescents, while the prevalence in the Swedish survey was lowest. This illustrates the cultural differences between countries, with higher social acceptability of alcohol drinking in minors in some areas, and the influence of more restrictive alcohol policies in others^(^ ^38^ ^)^. Italian children also showed a high propensity to consume sugar, while Swedish children had lowest scores for both sugar and fat propensity.

Despite this new evidence for an effect of childhood diet on later alcohol consumption, the identification of causal pathways remains difficult. In contrast to animal models it is difficult to determine the effects of single nutrients in human studies as they are often consumed together. As a candidate for a physiological link between consumption of diet in childhood and alcohol use, we mention the strong main effect of high fat propensity on alcohol consumption that was independent of sugar propensity. Mutual reinforcement of ethanol and fat consumption has been demonstrated in rodents^(^ ^16^ ^)^ and our findings may constitute a similar phenomenon in man. However, the large combined effect of high sugar and high fat propensity underlines the importance of taste preference^(^ ^14^ ^,^ ^17^ ^,^ ^18^ ^)^ that might predispose overconsumers of palatable foods to regular use of alcohol. Future studies on this cohort, using techniques ranging from genotyping to brain imaging, may shed more light on the addictive potential of palatable foods in adolescents and families.

Among the strengths of the present study is the comprehensive assessment of lifestyle factors of incipient users of alcohol in their different cultural contexts. The consistency of positive associations between sugar, fat and alcohol in spite of large between-country differences in consumption patterns underlines the robustness of the results. The longitudinal design with clear separation between measurements in childhood and in adolescence six years later strengthens the level of evidence that can be attributed to this observational study. The validity of food groups in the FFQ was reported at baseline^(^ ^23^ ^,^ ^25^ ^)^, except for the question on alcohol that was added at follow-up. However, the plausibility of the latter is supported by the concomitant correlation with higher impulsivity, smoking and parental alcohol consumption. A main study limitation is sample size, with just over 100 regular users of alcohol among the adolescents, restricting the possibility for subgroup and interaction analyses. Because attrition at follow-up was associated with overweight, lower well-being scores and lower parental education and income^(^ ^20^ ^,^ ^22^ ^)^, prevalence of alcohol use as well as of specific dietary patterns may be under- or overestimated. However, non-participation should not affect the size of associations between childhood diet and alcohol use in adolescence, as confirmed by adjustment for these measured confounding factors. Another important limitation is residual confounding due to unmeasured factors that could drive both ingestive behaviours we are studying. For instance, weak associations with parenting style could indicate that the variable was not specifically assessing supervision of dietary intake but capturing other aspects of family life. It is thus still possible that strict norms on both palatable foods and alcohol could explain their mutual association observed here. The lack of an age effect on alcohol consumption could be viewed as another limitation, possibly related to the fact that the age distribution is narrow compared with the entire course of adolescent development, when initiation to alcohol typically occurs^(^ ^39^ ^)^. Finally, we note that higher propensities for sugar and fat intake were associated with lower BMI *Z*-scores. While this direction of associations may be a sign of reverse causation, i.e. parents of overweight children under-reporting intakes of sugar and fat, we showed that weight status did not confound or modify any of the findings reported here.

## Conclusion

The present study explored the relationship between children’s consumption of sugar-rich and high-fat foods and their alcohol consumption in adolescence. The results suggest a positive association, which was only partly explained by important confounders measured in the study, or by sugar and fat consumption sustained into adolescence. The combined effect of fat and sugar might suggest habituation to palatability rather than effects of specific substances, although our method could not identify effects of single macronutrients. Covariation with poor mental health and lower parental income suggests that there are societal groups of particular vulnerability, in times when the abundance of cheap, unhealthy foods encourages unhealthy coping mechanisms. This calls for interventions increasing the awareness about the long-term impact of healthy eating early in life^(^ ^40^ ^)^, which may ultimately lead to legislative changes that influence reformulation, production, advertisement and distribution of unhealthy food products.

## Supplementary material

For supplementary material accompanying this paper visit https://doi.org/10.1017/S1368980018001829. click here to view supplementary material
