# Supplementary material for: Children’s propensity to consume sugar and fat predicts regular alcohol consumption in adolescence
Source: Public Health Nutr. 2018 Aug 24;21(17):3202–9. doi: 10.1017/S1368980018001829 (PMC6316354; doi:10.1017/S1368980018001829)
Supplement: Supplementary file 1 [file S1368980018001829sup001.pdf]

Table S1: Sample characteristics at baseline and differences by baseline exposures

		Sugar propensity		Fat propensity	
		≤ 24 %	> 24 %	≤ 24.6%	> 24.6 %
		(n = 1133)	(n = 1130)	(n = 1130)	(n = 1133)
Baseline covariates		(ref)		(ref)	
	Total	Mean (SD)	Mean(SD)	Mean (SD)	Mean (SD)
Age	2263	7.54 (0.78)	7.48 (0.76)	7.47 (0.77)	7.55 (0.76)
BMI z-score	2263	0.54 (1.19)	0.50 (1.17)**	0.69 (1.21)	0.36 (1.13)***
HRQoL	2053	39.8 (4.8)	39.2 (4.7)	39.9 (4.7)	39.1 (4.8)***
		N (%)	N (%)	N (%)	N (%)
Female sex	2263	569 (50%)	586 (52%)	591 (52%)	564 (50%)
High parental education	2237	616 (55%)	490 (44%)**	557 (50%)	549 (49%)
High parental income	2049	428 (42%)	334 (32%)	373 (37%)	389 (38%)
Parenting style: permissive	2140	117 (11%)	160 (15%)*	126 (12%)	151 (14%)
somewhat strict		543 (51%)	575 (53%)	539 (50%)	579 (54%)
strict (ref)		401 (38%)	344 (32%)	410 (38%)	335(31%)

* p < 0.05, ** p < 0.01, *** p < 0.001 (adjusted for age, sex, and country)

Table S2: Sample characteristics at follow-up and differences by outcome status

		**Weekly alcohol consumption at follow-up**	
Covariates at follow-up	Total	No (n = 2156, ref)	Yes (n = 107)
		Mean (SD)	Mean (SD)
Age	2263	13.37 (0.74)	13.36 (0.72)
BMI z-score	2263	0.52 (1.25)	0.69 (1.30)
Sugar propensity	2256	22.4 (9.9)	27.2 (10.6)***
Fat propensity	2258	27.5 (10.1)	31.6 (10.9)***
HRQoL	2059	38.4 (5.2)	37.2 (5.5)*
Impulsivity score	2063	24.7 (7.4)	28.4 (7.6)***
Parents' drinks per week	1627	1.5 (3.3)	2.6 (6.4)*
		N (%)	N (%)
Female sex	2263	1116 (52%)	39 (36%)**
Current smoking	2233	32 (2%)	13 (12%)***

* p < 0.05, ** p < 0.01, *** p < 0.001 (adjusted for age, sex, and country)

Table S3: Associations between sugar, and fat propensity at baseline and alcohol consumption at follow-up (RR, 95% CI), stratified by overweight (A) and by sex (B)

		**Categories of dichotomized sugar and fat propensities**			
		Low sugar, Low fat	High sugar, Low fat	Low sugar, High fat	High sugar, High fat
**A**	**Non-overweight children**				
	Alcohol consumers / total	12 / 431	17 / 419	15 / 358	33 / 485
	HR (95% CI)	1	1.38	1.38	2.30*
		(ref)	(0.66, 2.86)	(0.68, 2.81)	(1.21, 4.37)
	**Overweight children**				
	Alcohol consumers / total	7 / 188	4 / 95	6 / 153	13 / 134
	HR (95% CI)	1	1.13	1.43	3.03*
		(ref)	(0.41, 3.18)	(0.44, 4.67)	(1.27, 7.23)
**B**	**Boys**				
	Alcohol consumers / total	12 / 300	14 / 264	15 / 239	27 / 305
	HR (95% CI)	1	1.54	1.37	2.27*
		(ref)	(0.73, 3.22)	(0.64, 2.90)	(1.17, 4.41)
	**Girls**				
	Alcohol consumers / total	7 / 319	7 / 250	6 / 272	19 / 314
	HR (95% CI)	1	0.89	1.42	2.76*
		(ref)	(0.33, 2.42)	(0.54, 3.73)	(1.24, 6.15)

A: Adjusted for age, sex, and country

B: Adjusted for age and country

* $p < 0.05$

Table S4: Palatable food consumption at baseline and relative risk (95% CI) for alcohol consumption at follow-up by survey country.

		Palatable food consumption		
**Survey country**	Total	No[1]	Yes[1]	RR (95% CI)
**Italy**	465	17 / 342	17 / 123	2.78 (1.46, 5.27)**
**Estonia**	360	10 / 253	6 / 107	1.42 (0.53, 3.80)
**Cyprus**	357	15 / 284	8 / 73	2.07 (0.92, 4.70)
**Belgium**	45	0 / 18	2 / 27	-
**Sweden**	235	2 / 220	1 / 15	7.33 (0.70, 76.3)
**Germany**	328	3 / 187	5 /141	2.21 (0.54, 9.10)
**Hungary**	329	12 / 223	6 / 106	1.05 (0.41, 2.73)
**Spain**	144	2 / 117	1 / 27	2.17 (0.20, 23.0)
**All countries**	2263	61 / 1644	46 / 619	Common RR (95% CI)[2]
				2.01 (1.38, 2.92)***

[1] Number of alcohol consumers / total

[2] Breslow-Day test for homogeneity of odds ratios: p-value = 0.6
